# Identification of Critical Residues in the Carboxy Terminus of the Dopamine Transporter Involved in the G Protein βγ-Induced Dopamine Efflux

**DOI:** 10.3389/fphar.2021.642881

**Published:** 2021-03-24

**Authors:** José A. Pino, Gabriel Nuñez-Vivanco, Gabriela Hidalgo, Miguel Reyes Parada, Habibeh Khoshbouei, Gonzalo E. Torres

**Affiliations:** ^1^ Departamento de Medicina, Facultad de Medicina, Universidad de Atacama, Copiapó, Chile; ^2^ Centro de Bioinformática, Simulación y Modelado, Facultad de Ingeniería, Universidad de Talca, Talca, Chile; ^3^ Department of Pharmacology and Therapeutics, University of Florida College of Medicine, Gainesville, FL, United States; ^4^ Centro de Investigación Biomédica y Aplicada (CIBAP), Escuela de Medicina, Facultad de Ciencias Médicas, Universidad de Santiago de Chile, Santiago, Chile; ^5^ Facultad de Ciencias de la Salud, Universidad Autónoma de Chile, Talca, Chile; ^6^ Department of Neuroscience, University of Florida College of Medicine, Gainesville, FL, United States; ^7^ Department of Molecular, Cellular and Biomedical Sciences, CUNY School of Medicine at City College of New York, New York, NY, United States

**Keywords:** dopamine, dopamine transporter, efflux, amphetamine, g-protein βγ

## Abstract

The dopamine transporter (DAT) plays a crucial role in the regulation of brain dopamine (DA) homeostasis through the re-uptake of DA back into the presynaptic terminal. In addition to re-uptake, DAT is also able to release DA through a process referred to as DAT-mediated DA efflux. This is the mechanism by which potent and highly addictive psychostimulants, such as amphetamine (AMPH) and its analogues, increase extracellular DA levels in motivational and reward areas of the brain. Recently, we discovered that G protein βγ subunits (Gβγ) binds to the DAT, and that activation of Gβγ results in DAT-mediated efflux - a similar mechanism as AMPH. Previously, we have shown that Gβγ binds directly to a stretch of 15 residues within the intracellular carboxy terminus of DAT (residues 582–596). Additionally, a TAT peptide containing residues 582 to 596 of DAT was able to block the Gβγ-induced DA efflux through DAT. Here, we use a combination of computational biology, mutagenesis, biochemical, and functional assays to identify the amino acid residues within the 582–596 sequence of the DAT carboxy terminus involved in the DAT-Gβγ interaction and Gβγ-induced DA efflux. Our *in-silico* protein-protein docking analysis predicted the importance of F587 and R588 residues in a network of interactions with residues in Gβγ. In addition, we observed that mutating R588 to alanine residue resulted in a mutant DAT which exhibited attenuated DA efflux induced by Gβγ activation. We demonstrate that R588, and to a lesser extent F5837, located within the carboxy terminus of DAT play a critical role in the DAT-Gβγ physical interaction and promotion of DA efflux. These results identify a potential new pharmacological target for the treatment of neuropsychiatric conditions in which DAT functionality is implicated including ADHD and substance use disorder.

## Introduction

The regulation of brain dopamine (DA) levels is critical for the establishment and maintenance of physiological functions such as locomotor activity, cognitive processes, and motivated behaviors including reward, pleasure, and emotion (Iversen, 1971; Giros and Caron, 1993; Torres et al., 2003). Dysfunction of the DA system has been consistently implicated in a number of neurological and psychiatric disorders including: Parkinson’s disease, depression, schizophrenia, attention deficit hyperactivity disorder (ADHD), Tourette's syndrome, and substance use disorder (Torres et al., 2003; Bisaglia et al., 2013). The DA transporter (DAT) is a plasma membrane protein, which plays a crucial role in the regulation of extracellular DA. Deletion of the DAT gene in mice or pharmacological inhibition of the transporter leads to profound neurochemical changes characterized by an increase in extracellular DA (Gainetdinov et al., 2002). These results suggest that DA re-uptake through DAT plays a major role in both the termination of DA-mediated transmission, and in the maintenance of extracellular DA levels. In addition to the well-characterized re-uptake or DAT-mediated direct transport, the transporter can also function to release DA. This process, referred to as DAT-mediated DA efflux, is the means by which potent and highly addictive psychostimulants, such as AMPH and AMPH derivatives increase extracellular DA in motivational and reward areas of the brain (Sitte et al., 1998; Sulzer et al., 2005). AMPH is often therapeutically used at moderate doses for the treatment of ADHD and other attention disorders and thus, the psychostimulant is not exclusively associated with pathological behaviors (Heal et al., 2013).

Several years ago, we initiated a series of experiments to test the idea that endogenous mechanisms might lead to DAT-mediated DA efflux. We reasoned that if AMPH, an exogenous agent, is able to stimulate DA efflux through DAT, then endogenous mechanisms must exist to physiologically induce DAT-mediated efflux. Using a proteomic approach, we identified the G protein βγ subunit (Gβγ) as a DAT interacting protein, and we have performed a systematic characterization of this new protein-protein interaction. Heterotrimeric G proteins are key molecular switches in signal transduction pathways mediated by G-protein-coupled receptors (GPCRs). Activation of GPCRs by agonists results in the dissociation of Gα and Gβγ subunits, and the subsequent activation of effector systems (Oldham and Hamm, 2008). Previously, we have shown that Gβγ binds directly to a stretch of residues (residues 582–596) within the carboxy terminus (c-terminus) of DAT, and activation of Gβγ results in DAT-mediated efflux (Garcia-Olivares et al., 2013; Garcia-Olivares et al., 2017). Using the conditioned place preference (CPP) paradigm to evaluate motivational behavior, and measuring locomotor activity we have shown that activation of Gβγ enhances, whereas inhibition reduces AMPH-evoked extracellular DA, locomotor activity and CPP (Mauna et al., 2019). These data suggest that signaling mechanisms involving Gβγ activation and binding to DAT represent an endogenous physiological mechanism leading to DAT-mediated efflux and produces a behavioral phenotype.

To further characterize the DAT-Gβγ interaction, we have now investigated the specific residues within the 582–596 sequence of the DAT c-terminus responsible for Gβγ binding and DAT-mediated efflux. Using *in-silico* estimations of protein-protein interactions and site-directed mutagenesis, we provide evidence for key residues within the c-terminus of DAT in Gβγ binding and DAT-mediated efflux.

## Materials and Methods

### Molecular Structures of Human DAT and Gβγ

**FIGURE 1:**
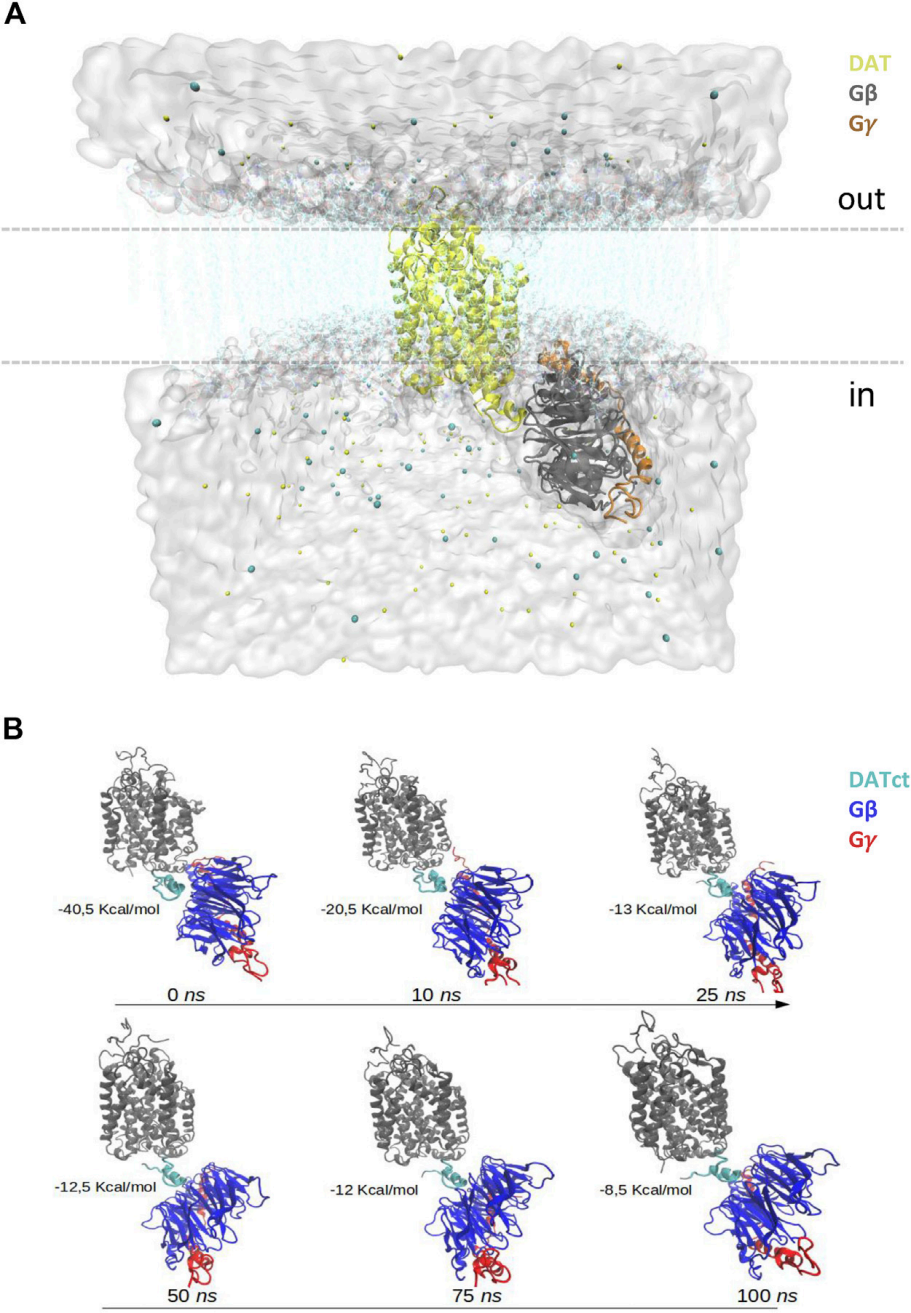
*In silico* analysis of the DAT-Gβγ complex. **(A)** Schematic representation of the DAT-Gβγ complex. The white surface represents water, the cyan lines denote the bilayer lipid membrane, cyan and yellow spheres indicate ions. DAT is presented in yellow. Gβ is shown in grey and Gγ in orange. **(B)** Contact frequency between DAT and Gβγ among 100 *ns* of dynamics simulation.

As previously described (Penmatsa et al., 2013), a homology model of the human DAT was built using the crystallographic structure of the DAT from *Drosophila melanogaster* as template (PDBid 4M48; 2.96Å). The human Gβγ complex was extracted from the molecular assembly of the µ-opioid receptor-G protein (PDBid 6DDE, 3.5Å; Koehl et al., 2018). To form the initial DAT-Gβγ complex, both structures were submitted to the ClusPro server (Kozakov et al., 2017) and the protein-protein docking pose whose conformational orientation agrees with the transmembrane features of DAT and cytoplasmic orientation of Gβγ was selected (Clapham, 1996; Oldham and Hamm, 2008). This complex, was embedded in a bilayer membrane of hydrated palmitoyl-oleyl-phosphatidyl-choline (POPC), entirely solvated in a water box (TIP3: “3-point rigid water model” type), and ions were added to create an overall neutral system in approximately 0.2 M NaCl (Figure 1A). The final system was subjected to a structural minimization and molecular dynamics (MD) simulations of 100 ns using the Nanoscale Molecular Dynamics (NAMD) software (Nelson et al., 1996).

### *In Silico* Analysis of the Interaction of DAT and Gβγ

The contact frequency between DAT and Gβγ during the 100 ns of dynamic simulation was obtained and analyzed using our own tool command language (TCL) scripts which run in complement with the Visual Molecular Dynamics (VMD) software (Humphrey et al., 1996). This analysis is performed considering the side-chain atoms of the residues that are located up to 4Å between DAT and Gβγ (Figure 1B). In addition, representative frames of the simulation of DAT-Gβγ (0, 10, 25, 50, 75 and 100 ns) were analyzed using the Protein-Protein interactions in Macromolecular Assembly (PIMA) and Protein-Protein Interfaces and Prediction of Residue Hotspots (PPCheck) web servers (Sukhwal and Sowdhamini, 2013). These tools are used to identify the binding interactions between critical residues on the interface zone of the DAT-Gβγ complex. For the free energy of binding calculations, these tools utilize a threshold distance of 7–10Å between the residues located in the protein-protein interface zone. In this zone, all the hydrogen bonds, electrostatic and Van der Waals contributions are considered as total energy.

### Cell Culture, Transfections, and Treatments

Chinese hamster ovary (CHO) cells were purchased from American Type Culture Collection. CHO cells were cultured in Ham's F-12 medium supplemented with 10% fetal bovine serum (FBS, Gibco), 1 mM L-glutamine, 100 U/ml penicillin and 100 μg/ml streptomycin (Sigma-Aldrich). The human DAT cDNA was cloned into pEYFP-C1 vector and used to transfect CHO cells with Lipofectamine 2000 (Invitrogen). For stable transfections, DAT-expressing single clones (CHO-DAT cells) were selected with G418 (Gibco), verified by DAT immunoblot, and maintained in Ham’s F-12 media containing 0.5 mg/ml G418. Site–directed Mutagenesis (QuickChange II™, Agilent) was used to create single amino acid changes (alanine substitutions) within the c-terminus of DAT (DATct) between residues 587–590 (F587-R588-E589-K590). DAT function was analyzed via DA uptake and efflux experiments, which were conducted 24–48 h after transfection in 24-well plates. CHO-DAT cells or cells expressing DAT mutants were seeded in 24-well plates coated with poly-d-Lysine (Sigma-Aldrich). For the treatments, cells were incubated with different drug conditions: 1) Amphetamine (AMPH: DA-releaser, Sigma-Aldrich) ranging from 0.1 to 100 μM, 2) mSIRK (Gβγ activator: myr-SIRKALNILGYPDYD, EMD Chemicals) ranging from 1.0 to 100 μM, 3) 40 μM scramble-mSIRK (scr-mSIRK: myr-SLYRLISLAPRGDYD, NeoBioScience, negative control), and 4) mSIRK + 10 μM GBR12935 (DAT blocker, Sigma-Aldrich). Drugs were prepared from individual 10 mM stocks in efflux buffer (4 mM Tris base, 6.25 mM HEPES, 120 mM NaCl, 5mM KCl, 1.25 mM MgSO_4_, 0.57 mM ascorbic acid, 5.6 mM glucose, 1 mM tropolone, pH 7.4) or DMSO. The control cells were incubated with efflux buffer containing 0.1% DMSO (vehicle) or efflux buffer. After the incubations, DAT-mediated DA uptake or efflux were tested in CHO-DAT and CHO-DAT mutant cell lines.

### [^3^H]-DA Uptake Assay

[^3^H]-DA uptake measurements in CHO cells transfected with WT DAT or mutated DAT were used to normalize DA efflux relative to the total amount of intracellular [^3^H]-DA according a previous protocol (Egaña et al., 2009). Briefly, cells seeded in 24-well plates were loaded for 20 min at 37°C with 250 μl of 60 nM of [^3^H]-DA (3,4-[7-^3^H] dihydroxyphenylethylamine, 27.8 Ci/mmol; Perkin Elmer, Waltham, CA, United States) in uptake buffer (4 mM Tris base, 6.25 mM HEPES, 120 mM NaCl, 5 mM KCl, 1.25 mM CaCl_2_, 1.25 mM MgSO_4_, 0.57 mM ascorbic acid, 5.6 mM glucose, 1 mM tropolone, pH 7.4). After loading with [^3^H]-DA, cells were washed with 1 ml ice-cold efflux buffer (4 mM Tris base, 6.25 mM HEPES, 120 mM NaCl, 5 mM KCl, 1.25 mM MgSO_4_, 0.57 mM ascorbic acid, 5.6 mM glucose, 1 mM tropolone, pH 7.4). 4 wells were solubilized in 0.4 ml of 1% SDS and incubated at room temperature for 1 h. After incubation, the solution inside the wells containing the intracellular [^3^H]-DA was collected and transferred to scintillation vials filled with 4 ml scintillation counting fluid (RPI Bio-safe II™). Counts per min (cpm) were obtained using a LS-6500 Liquid scintillation counter (Beckman Coulter, Brea, CA, United States). The remaining 20 wells were used for [^3^H]-DA efflux experiments.

### [^3^H]-DA Efflux Assay

The conditions to test DAT-mediated efflux in CHO cells expressing either wild-type **(**WT) DAT or mutated DAT have been described previously (Garcia-Olivares et al., 2017). Briefly, cells seeded in 24-well plates were loaded for 20 min at 37°C with 250 μl of 60 nM of [^3^H]-DA in uptake buffer. After loading with [^3^H]-DA, cells were washed with 1 ml ice-cold efflux buffer and then incubated for 10 min at 37°C with 500 μl of efflux buffer in the absence or presence of different drug conditions. After the 10min efflux assay period, the released [^3^H]-DA was collected from the extracellular medium, transferred to scintillation vials filled with 4 ml scintillation counting fluid (RPI Bio-safe II™), and cpm were obtained. Finally, to quantify the efflux in each plate, [^3^H]-DA efflux measurements were normalized to the total [^3^H]-DA uptake after the 20 min preload period. The fractional [^3^H]-DA efflux ([^3^H]-DA efflux/[^3^H]-DA uptake) was used to study the functional effects of DAT mutations. Efflux is expressed as a percentage, relative to baseline levels of extracellular [^3^H]-DA in the absence of treatment.

### Amperometry

HEK-YFP-DAT cells were preloaded with DA as described previously (Goodwin et al., 2009; Roth et al., 2013; Sambo et al., 2018). Briefly, the cells were washed twice with external solution (130 mM NaCl, 1.3 mM KCl, 2.2 mM CaCl_2_, 1.2 mM MgSO_4_, 1.2 mM KH_2_PO_4_, and 10 mM HEPES, pH 7.4) containing 10 mM D-glucose. The cells then were incubated with the external solution containing 1 μM DA, 100 μM pargyline, 10 μM tropolone, and 100 μM ascorbic acid for 20 min at 37°C. Following three washes, a carbon fiber electrode (ProCFE; 5 μm diameter; Dagan Corp.) was positioned opposed to the plasma membrane of a cluster of cells that expressed YFP-DAT. The cells were held at + 700 mV with respect to the bath ground (a potential greater than the oxidizing potential of DA) to measure DA efflux via oxidation reactions. The amperometric electrode measures the electrical currents (pA) as a result of released DA oxidation. Amperometric currents were recorded using Axopatch 200B with a lowpass Bessel filter set at 1000 Hz and digitally filtered off line at 10 Hz before analysis. The DAT-mediated DA efflux was defined as baseline amperometric current minus the current after the bath application of 10 μM nomifensine, a DAT and norepinephrine transporter inhibitor. The Gβγ-induced DAT-mediated DA efflux was defined as baseline amperometric current minus the current after the bath application of 10 μM mSIRK peptide (Gβγ activator). Data were acquired by averaging 10 s intervals of current directly before nomifensine application (baseline current) and 20 min following nomifensine application (nomifensine current). For Gβγ inhibition, cells were pretreated before amperometric recording for 20 min in external solution containing a 20 μM concentration of Gallein, a known inhibitor.

### Western Blot

40 µg protein samples were run in an SDS-PAGE 10% gel electrophoresis, and transferred to nitrocellulose or PVDF membranes. The membrane was blocked for 1 h with 5% milk in TBST (25 mM Tris/TrisHCl, 130 mM NaCl, 2.7 mM KCl, 0.2% Tween 20). To identify the DAT, the rat anti-DAT MAB369 antibody was used (1:1000, 2 h at room temperature, Millipore). Membranes were washed with TBST 3 times by hand and 3 times for 5 min with gentle agitation. An anti-rat HRP-conjugated secondary antibody (1:5000, 1 h at RT, Jackson Immunoresearch Lab) was used to detect DAT immunoreactive bands. Membranes were again washed with TBST 3 times by hand and 3 times for 5 min with gentle agitation. Immunoreactive bands were visualized using either Clarity Western ECL Substrate (1705060, Bio-Rad Laboratories) or Clarity Max Western ECL Substrate (1705062, Bio-Rad Laboratories).

### Biotinylation Assay

CHO cells were washed with PBS and then incubated with gentle agitation for 30 min at 4°C with 1 ml of 1.5 mg/ml sulfo-NHS-SS-biotin prepared in biotinylation buffer (150 mM NaCl, 2 mM CaCl_2_, 10 mM triethanolamine, pH 7.8). Incubating the cells for additional 10 min with 50 mM glycine in PBS quenched the reaction. Cells were then washed with PBS and lysed at 4°C for 1 h with lysis buffer D (20 mM HEPES, 125 mM NaCl, 10% Glycerol, 1 mM EDTA, 1 mM EGTA, pH 7.6) containing 1% Triton x-100 and protease inhibitors. Cell lysates (500 μl) were then divided into three aliquots: 1) 100 μl for protein measurement, 2) 80 μl for SDS-PAGE 10% gel electrophoresis SDS (total fraction sample), and 3) the remaining 320 μl to obtain the biotinylated fraction containing the proteins in the cell surface. The biotinylated proteins were precipitated via incubating the biotinylated protein lysates for 1 h at 4°C with 80 μl of ultralink-immobilized avidin beads (50% slurry in lysis buffer, Pierce). Samples were subsequently centrifuged at 2500 x g for 2 min, the supernatant was discarded, and the beads were washed with lysis buffer. Finally, 40 μl of 2X laemmli sample buffer were added to each protein sample in order to analyze DAT expression by SDS-PAGE on 10% Tris-HCl polyacrylamide gels and immunoblotting using antibodies against DAT (anti-DAT MAB369), and an HRP-conjugated secondary antibody.

### Data Analysis

DA uptake and efflux are expressed as the mean ± SEM. The values of [^3^H]-DA released were analyzed using one-way ANOVA with Tukey’s multiple comparison test. DA release induced by different doses of AMPH and the Gβγ activator mSIRK were analyzed in CHO cells expressing either wild-type (WT) DAT or mutated DAT using two-way ANOVA with Bonferroni post-test. Significance was set at *p* < 0.05.

## Results

### *In silico* Analysis of the DAT-Gβγ Complex Predicts that Specific Residues within the Carboxy Terminus of DAT Interact with Gβγ Subunits

**FIGURE 2:**
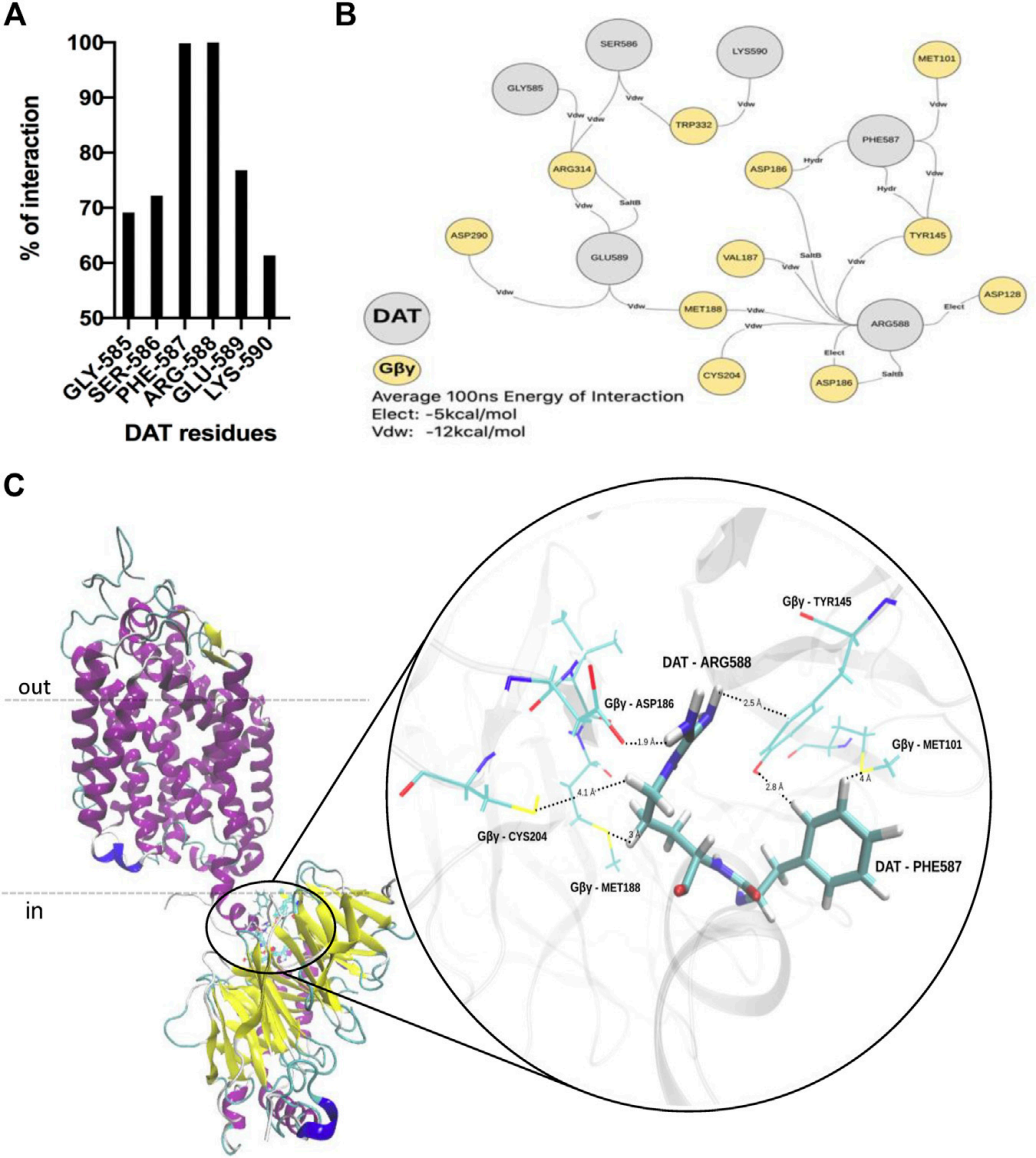
F587 and R588 within the carboxy terminus of DAT are involved in the interaction between DAT and Gβ. **(A)** *In-silico* protein-protein interaction analyses predict that residues PHE587 (F587) and ARG588 (R588) located on DAT are the most frequent residues that interact with Gβ. **(B)** F587 and R588 stabilize the DAT-Gβ complex via Van der Waals, hydrogen bonds and electrostatic interactions with several residues located on Gβ. **(C)** Model showing the most frequent interactions that stabilize the DAT-Gβ complex.

Our group has previously shown via co-immunoprecipitation and *in vitro* protein-protein interaction assays that the c-terminus of human DAT (DATct) interacts physically with Gβγ subunits (Garcia-Olivares et al., 2013, Garcia-Olivares et al., 2017). To facilitate the analysis, we ran protein-protein docking and MD simulation to model the architecture of the DAT-Gβγ complex and to identify any specific chemical interactions and critical residues on the protein-protein interaction zone. Accordingly, our *in silico* protein-protein docking data along with the contact frequency between DAT and Gβγ analysis (100 ns of dynamics simulation) indicate that there is a predominant interaction between the c-terminus of DAT and Gβ of the Gβγ complex. These results support our previous findings showing a physical association between the c-terminus of DAT and Gβγ subunits using biochemical assays with purified proteins (Garcia-Olivares et al., 2013). The data also validate the use of the model in the characterization of the complex conformation (Figure 1). Using the PIMA and PPCheck servers we analyzed representative frames of the MD simulation of DAT-Gβγ complex (0 ns, 10 ns, 25 ns, 50 ns, 75ns and 100 ns) to understand the critical residues and specific chemical interactions between residues that stabilize the protein-protein interaction zone (Figure 2). This analysis predicts that during the 0–100 ns period the lateral chains of two critical residues within the c-terminus of DAT (F587 and R588) are located in a distance below 4Å and suggests that these residues could be involved in the interaction between DAT and Gβγ (Figure 2A, 100% probability of interaction). Further, we observed that DAT-F587 interacts with: 1) Gβ-MET101 and TYR145 via Van der Waals interactions; and 2) Gβ-TYR145 and Gβ-ASP186 via hydrogen bonds. On the other hand, DAT-ARG588 interacts with: 1) Gβ-ASP186 and Gβ-ASP128 via electrostatic interactions; 2) Gβ-TYR145, Gβ-MET188, Gβ-VAL187, and Gβ-CYS204 via Van der Waals interactions; and 3) Gβ-ASP186 via a potential salt bridge (Figures 2B,C). These results provide a structural framework, which predicts stabilization of the DAT-Gβγ complex with specific residues the c-terminus of DAT.

### Gβγ Activity Modulates DA Efflux Through DAT

**FIGURE 3:**
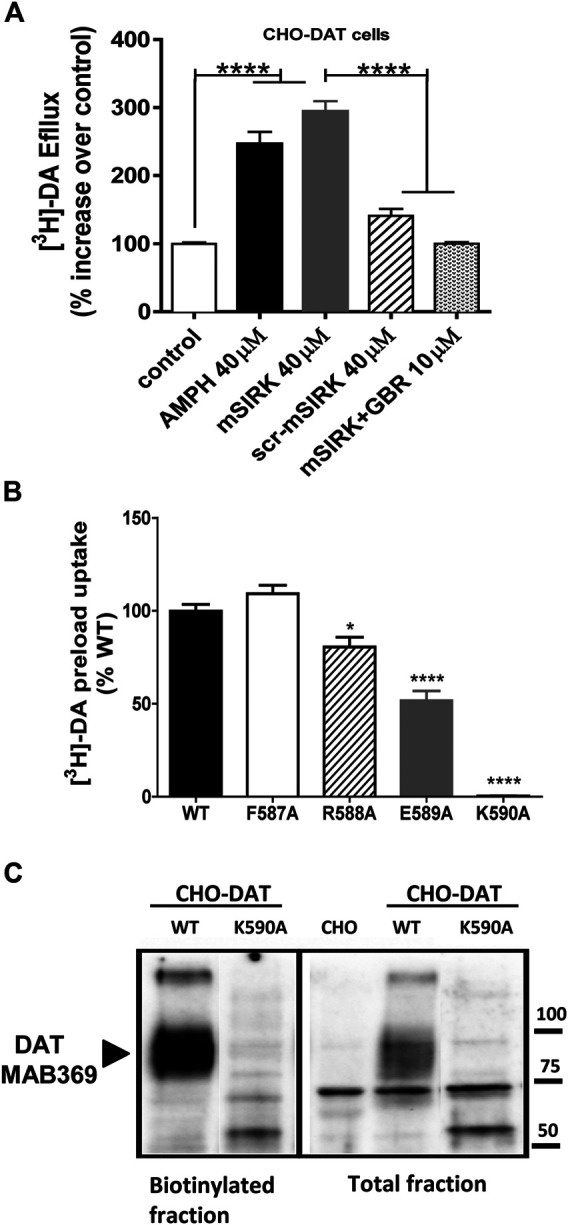
Mutations in the FREK sequence of DAT differentially alter plasma membrane expression and transporter function. **(A)** [^3^H]-DA efflux assays in CHO-DAT cells. Results are expressed as the mean ± SEM (*N* = 12). *p < 0.05, one-way ANOVA with Tukey’s multiple comparison test. **(B)** [^3^H]-DA uptake in CHO-DAT and CHO cells expressing mutated DAT. Results are expressed as the mean ± SEM (*N* = 6). *p < 0.05; ****p < 0.0001, one-way ANOVA with Dunnett’s multiple comparison test. **(C)** Cell surface biotinylation assays in CHO-DAT and CHO cells expressing the K590 mutant.

To begin exploring the role of specific residues within the c-terminus of DAT predicted by the *in-silico* analysis, we first established the conditions for DA efflux experiments in CHO cells expressing WT DAT (CHO-DAT, Figure 3A). In CHO-DAT cells preloaded with [^3^H]-DA, AMPH induces a significant increase in DA efflux (2.6-fold above control, Figure 3A). As reported previously, the specific Gβγ activator mSIRK induces an increase in DA efflux similar to the effect elicited by AMPH (2.8-fold above control Figure 3A). The scramble peptide (scr-mSIRK) used as a negative control does not change DA efflux significantly. Importantly, the Gβγ-dependent DA efflux observed with mSIRK is blunted when cells are pre-incubated with the DAT blocker GBR12935 (Figure 3A). These results confirm that activation of Gβγ signaling promotes DA efflux through DAT (Garcia-Olivares et al., 2017) and establish the conditions to examine the functional effect of DAT mutants on Gβγ-induced DA efflux through the transporter.

### Mutations in Key Residues Within the DAT-Gβγ Binding Site Differentially Affect Plasma Membrane Expression and Uptake Function

The *in-silico* protein-protein docking analysis predicts that residues F587 and R588 of the c-terminus of DAT are important for the stabilization of the DAT-Gβγ complex through a network of interactions with Gβ. Further, the model predicts that residues E589 and K590 have a minor role in the interaction (Figure 2A). To experimentally test the model, we performed single alanine substitutions by site-directed mutagenesis and mutated residues F587, R588, E589, and K590 to alanine residues. These mutant transporters were transfected in CHO cells and the ability of these mutant transporters to evoke efflux after Gβγ activation was compared to the WT DAT. In order to measure transporter-mediated efflux, the mutant transporters must be able to be expressed at the cell surface and uptake DA such that the cells can be pre-loaded with the transmitter for our efflux assays. The F587A and R588A DAT mutants showed similar uptake activity compared to WT DAT while the E589A mutant exhibited a 50% reduction of activity (Figure 3B). Thus, these results demonstrate that F587A, R588A, and E589A are all expressed at the cell surface and are able to uptake DA. In contrast, the K590A did not exhibit transporter activity above background (Figure 3B). To determine if the K590A mutant was expressed at the plasma membrane, we performed cell surface biotinylation experiments. The mature form of WT DAT, ∼ 80kDa, is detected in both the total and the biotinylated fractions (Figure 3C). However, the K590A mutant did not result in a mature form of DAT but rather was expressed predominantly as a non-mature form, ∼60 kDa. Therefore, the lack of uptake activity by the K590A mutant can be explained by the lack of cell surface expression of the DAT mutant.

### F587 and R588 Play an Important Role in the Gβγ-Induced DAT-Mediated DA Efflux

**FIGURE 4:**
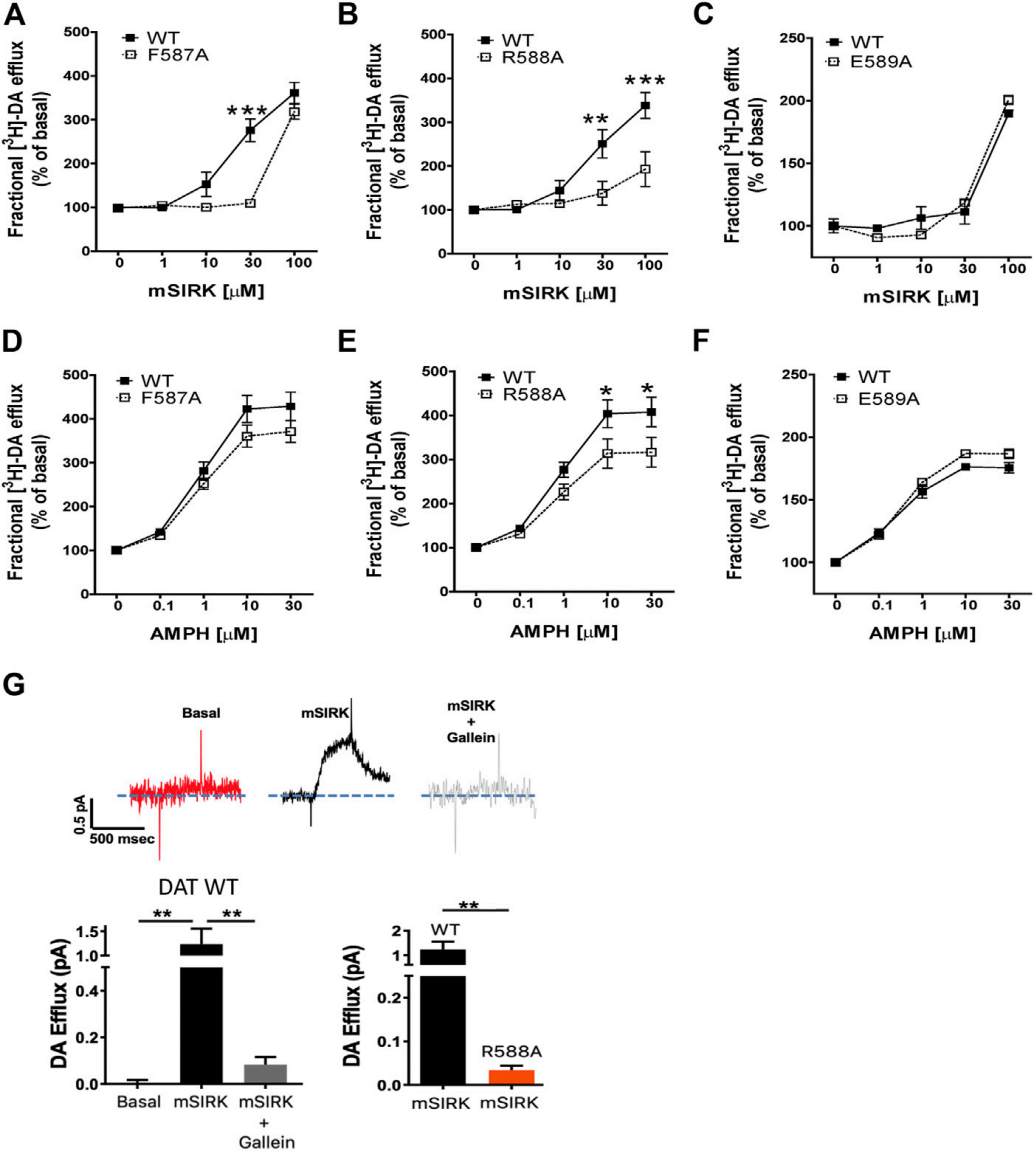
Residues F587 and R588 of DAT modulate DA efflux induced by AMPH and the Gβγ activator mSIRK. **(A–C)** Fractional DA efflux induced by Gβ**γ** signaling. **(D–F)** Fractional DA efflux induced by AMPH. Results are expressed as the mean ± SEM (*N* = 6). *p < 0.05, two-way ANOVA with Bonferroni post-test. **(G)** Amperometry recordings showing the outward current attributed to the release of preloaded DA from cells expressing WT DAT and DAT R588A. Results are expressed as the mean ± SEM (*N* = 4). **p < 0.01; ***p < 0.001, one-way ANOVA with Tukey’s multiple comparison test. For the comparison between DAT WT and DAT R588A, results are expressed as the mean ± SEM (*N* = 4). ***p < 0.001, unpaired *t*-test.

We performed [3H]-DA efflux assays on CHO cells stably transfected with WT DAT and each functional DAT mutant (F587A, R588A, and E589A). Here, we tested and compared the effect of Gβγ activation with mSIRK (Figures 4A–C) with that of AMPH (Figures 4D–F). DA efflux was analyzed as a fractional efflux, in which DA efflux is normalized to DA loading for each DAT mutant or WT DAT in the 20 min preload period. Results are expressed as a percentage of baseline (without AMPH or mSIRK). As expected, AMPH as well as mSIRK induced a dose–dependent increase in DA efflux in cells transfected WT DAT. As predicted by the model, the E589A mutant showed similar functional DAT dynamics as WT DAT regarding AMPH- and mSIRK-induced DA efflux (Figures 4C,F). The F587A and R588A mutations generated a small decrease in AMPH-induced DA efflux when compared with the WT transporter; 15 and 23% reduction, respectively (Figures 4D,E). Interestingly, the R588A and F587A mutations lead to a larger reduction of DA efflux when exposed to mSIRK as compared to WT DAT (46% reduction in R588A mutants; Figures 4A,B). Amperometry was used on cells containing the R588A mutation to confirm the attenuated effect of mSIRK-induced efflux. In WT DAT cells, the application of mSIRK elicited a robust outward current attributed to the release of preloaded DA, which was blocked in the presence of the Gβγ blocker gallein. As seen with efflux experiments using [^3^H]-DA, DA efflux induced by activation of Gβγ with mSIRK in cells expressing R588A DAT was dramatically attenuated (Figure 4G). Taken together, our experimental data validate the *in-silico* protein-protein docking model, and strongly suggests that residues F587 and in a larger degree R588, located in the c-terminus of DAT, play a pivotal role in DA efflux induced by Gβγ signaling.

## Discussion

Recently, our laboratory discovered that Gβγ subunits interact physically with DAT, and this interaction is associated with DAT-mediated DA efflux in cells in culture, DA neurons in culture, striatal brain slices, and *in-vivo*. Specifically, we have shown that Gβγ activation promotes DA efflux through DAT, which resembles the action of AMPH on the DAT (Garcia-Olivares et al., 2013, Garcia-Olivares et al., 2017). Additionally, we have demonstrated that Gβγ signaling modulates AMPH actions both *in vitro* as well as *in vivo* (Mauna et al., 2019). These findings strongly suggest that the release of DA by DAT is a mechanism that could be endogenously activated through G proteins, and is not solely observed in the presence of psychostimulants.

In the present study, we identified specific residues within the c-terminus of DAT involved in Gβγ binding and DAT-mediated efflux. Previously, we have established that the physical interaction between DAT and Gβγ involves the sequence 582–596 located on the c-terminus of the transporter (Garcia-Olivares et al., 2017). We hypothesized that changes in specific residues within the 582–596 sequence of DAT would result in altered Gβγ-mediated DA efflux. Our *in silico* MD protein-protein docking analysis predicted that residues F587 and R588 are important for the stabilization of the DAT-Gβγ complex through electrostatic, hydrogen bonds, and Van der Waals interactions with several Gβ residues (Figures 1,2). Thus, these predictions provide a network of residues involved in the DAT-Gβγ complex. Furthermore, based on our *in-silico* predictions, we performed site–directed mutagenesis to generate single alanine substitutions in a critical sequence (F587-R588-E589-K590, “FREK”) of the c-terminus of DAT. Our data showed that the mutant K590A is not detected at the plasma membrane and therefore, it does not result in a functional transporter. Thus, the K590 residue may be essential for DAT processing and/or trafficking to the cell membrane. These data are consistent with previous results demonstrating the critical role of DAT residues 587–596, corresponding to the sequence FREKLAYAIA in transporter trafficking (Melikian and Buckley, 1999; Holton et al., 2005; Gabriel et al., 2013). Overall, these data suggest that this region is critical for protein-protein interactions, not only regulating activity such as Gβγ, but also interactions regulating transporter trafficking. Thus, the FREKLAYAIA sequence may define a “hotspot” in the carboxy terminus of DAT for protein-protein interactions that modulate the transporter.

Our functional experiments in which we analyzed DA efflux in CHO cells transfected with DAT are consistent with our computational predictions, and support the conclusion that R588 and to a lesser extent F587 are relevant for the Gβγ-dependent DAT-mediated DA efflux (Figure 4). To a lesser extent, these residues are also involved in the AMPH-induced DA efflux, which supports the idea that the AMPH-induced DA efflux is in part, mediated by Gβγ (Mauna et al., 2019). Our computational model predicts that residue F587 in DAT interacts with three residues in Gβ (MET101, TYR145, ASP186), whereas R588 in DAT interacts with at least seven residues in Gβ (MET101, ASP128, TYR145, ASP186, VAL187, MET188, and CYS204), and the network of interactions is critical for binding and stabilization of the protein complex. Interestingly, a recent publication by Gulati et al. (2018) demonstrated that a specific nanobody, Nb5, binds to a region on the Gβγ dimer shared with other Gβγ regulatory proteins, including phosducin and G protein-coupled receptor kinase 2. Here, the authors demonstrated that Nb5 interferes with the ability of Gβγ to associate with G protein-coupled inwardly rectifying potassium (GIRK) channels, suppressing Gβγ-regulated GIRK signaling, while leaving Gα-mediated signaling intact. Gβγ-Nb5 binding involves several Gβ residues (MET101, TYR145, ASP186, MET188, and CYS204) those of which are identical residues predicted by our computational model to bind with DAT. Thus, this study supports the contention that Gβγ contains a binding interphase or structural “hot-spot” shared by effector molecules, including DAT. Future studies from our lab and others will examine the contribution of predicted Gβ residues, and the effect of Nb5 in Gβγ-dependent DAT-mediated DA efflux.

We propose a mechanism for endogenous DAT-mediated DA efflux via the formation of a DAT-Gβγ complex. This involves approximately seven residues from Gβ (MET101, ASP128, TYR145, ASP186, VAL187, MET188, and CYS204) and two residues from the FREK sequence in the carboxy terminus of DAT (F587 and R588). Several studies have examined the role of protein-protein interactions in DAT function (Kristensen et al., 2011; Sager and Torres, 2011). Some of these interactions include syntaxin1A (Binda et al., 2008), PKCβ (Johnson et al., 2005), calmodulin kinase IIα (CamKIIα) (Fog et al., 2006) and flotillin-1 (Cremona et al., 2011). In addition to the role of synatxin1A in DAT membrane trafficking, it also promotes AMPH-induced dopamine efflux through a mechanism presumably involving phosphorylation of the transporter. The interaction between DAT and syntaxin1A was mapped to the amino terminus of DAT, suggesting the presence of a network of protein-protein interactions involving several DAT domains (Binda et al., 2008). Similarly, PKCβ may play a role in AMPH-induced DA efflux through DAT (Johnson et al., 2005). Inhibition of PKCβ abolishes DA efflux in brain slices, and the ability of AMPH to induce DA efflux was significantly enhanced in heterologous cells overexpressing PKCβ. These effects of PKCβ were not due to changes in DAT cell surface expression, but rather through changes in the intrinsic activity of the transporter. CamKIIα, has also been observed to bind with DAT and regulate AMPH-induced DA efflux (Fog et al., 2006). The interaction site between CamKIIα and DAT was mapped to the carboxy terminus of the transporter, and the data support a model in which the binding of CamKIIα to the carboxy terminus induces phosphorylation of serine residues at the amino terminus of DAT. Substitution of these serine residues at the amino terminus abolished the effect of AMPH on DAT efflux (Khoshbouei et al., 2004; Fog et al., 2006). A functional link has been established between CamKIIα activation and the interaction between syntaxin1A with the related norepinephrine (NET) and serotonin (SERT) transporters (Dipace et al., 2007; Binda et al., 2008; Ciccone et al., 2008) as inactivation of CamKIIα decreases the amount of syntaxin1A co-immunoprecipitated with NET. One may speculate that an interaction between Gβγ to the carboxy terminus of DAT promotes the concerted binding of a network of proteins including CamKIIα, syntaxin1A, and/or PKCβ to induce phosphorylation of the transporter’s amino terminus and promote DA efflux. Collectively, these data suggest a model in which substrate efflux is regulated by a complex interplay involving several protein−protein interactions.

We further hypothesize that the formation of this DAT-Gβγ complex promotes DA efflux through conformational changes inside of a high affinity-binding pocket near the primary substrate-binding site, S1, on DAT. This proposed mechanism is similar to the DAT-mediated DA efflux induced by the binding of AMPH. These conformational changes on DAT observed after AMPH binding have been suggested in a previous report and review (Kristensen et al., 2011; Cheng et al., 2015). More specifically, binding of AMPH to the S1 on DAT promotes a conformational change from the outward-facing open (OFo) to the outward facing closed (OFc*) state. This structural change is associated with the formation of a salt bridge between residues R85 and D476 (lateral chain distance between residues below 2Å during the simulation) or with the movement of transmembrane domains on DAT (Cheng et al., 2015). Interestingly, we have confirmed these structural changes in a preliminary simulation of DAT-Gβγ complex (Supplementary Figure S1). These preliminary findings will guide us to the develop of future computational simulations and mutational analysis like the coarse-grained molecular dynamics simulation method (Negami et al., 2020), that allow us to simulate and to determine conformational changes on DAT in the potential transition from OFo to OFc* that could be induced by the formation of the DAT-Gβγ complex. In addition, future experiments should test the contribution of specific residues in Gβ involved in the interaction with DAT as predicted by the model. Our data identify residues F587 and R588 as critical for DAT-mediated DA efflux might provide a new pharmacological target for the identification of compounds that can exploit the DAT-mediated DA efflux mechanism for the treatment of neuropsychiatric conditions including attention disorders and substance use disorder.

## Data Availability

The datasets presented in this study can be found in online repositories. The names of the repository/repositories and accession numbers can be found below: https://gitlab.com/nunezvivancogabriel/datgbg, 22877308.
