# Supplementary material for: Identification of Critical Residues in the Carboxy Terminus of the Dopamine Transporter Involved in the G Protein βγ-Induced Dopamine Efflux
Source: Front Pharmacol. 2021 Mar 24;12:642881. doi: 10.3389/fphar.2021.642881 (PMC8025876; doi:10.3389/fphar.2021.642881)
Supplement: Supplementary file 1 [file datasheet1.pdf]

Supplementary table S1

Table S1.1. Statistical results for data depicted in Figure 3A. * denotes statistically significant. One-way ANOVA with Tukey's multiple comparison test. Data sets analyzed: control, amphetamine (AMPH), mSIRK, scr-mSIRK, mSIRK + GBR12935.

ANOVA summary	F (DFn, DFd)		P value		
Treatment<br>Significant diff. among means (P < 0.05)? = yes	F(4,55) =60.14		p<0.0001****		
**Tukey's multiple comparisons test**	**Mean Diff.**	**95.00% CI of diff.**	**Significant?**	**Summary**	**Adjusted P Value**
control vs. scr-mSIRK 40mM	-41,01	-86.83 to 4.809	No	ns	0,0999
control vs. mSIRK 40mM	-194,3	-240.2 to -148.5	Yes	****	<0.0001
control vs. mSIRK+GBR 10mM	-0,2177	-46.04 to 45.6	No	ns	>0.9999
control vs. AMPH 40mM	-146,7	-192.6 to -100.9	Yes	****	<0.0001
scr-mSIRK 40mM vs. mSIRK 40mM	-153,3	-199.1 to -107.5	Yes	****	<0.0001
scr-mSIRK 40mM vs. mSIRK+GBR 10mM	40,79	-5.027 to 86.62	No	ns	0,1029
scr-mSIRK 40mM vs. AMPH 40mM	-105,7	-151.5 to -59.9	Yes	****	<0.0001
mSIRK 40mM vs. mSIRK+GBR 10mM	194,1	148.3 to 239.9	Yes	****	<0.0001
mSIRK 40mM vs. AMPH 40mM	47,61	1.789 to 93.43	Yes	*	0,0379
mSIRK+GBR 10mM vs. AMPH 40mM	-146,5	-192.3 to -100.7	Yes	****	<0.0001

Table S1.2. Statistical results for data depicted in Figure 3B. * denotes statistically significant. One-way ANOVA with Dunnett's multiple comparison test. Data sets analyzed: wild type (WT), and the following DAT mutant residues: F587A, R588A, E589A, and K590A.

ANOVA summary	F (DFn, DFd)		P value		
Treatment<br>Significant diff. among means (P < 0.05)? = yes	F(4,31) =63.24		p<0.0001****		
**Dunnett's multiple comparisons test**	**Mean Diff.**	**95.00% CI of diff.**	**Significant?**	**Summary**	**Adjusted P Value**
WT vs. F587A	-7239	-19855 to 5377	No	ns	0,3978
WT vs. R588A	14830	2214 to 27446	Yes	*	0,0173
WT vs. E589A	37071	24455 to 49687	Yes	****	0,0001
WT vs. K590A	76402	60950 to 91853	Yes	****	0,0001

Table S1.3. Statistical results for data depicted in Figures 4A-F. * denotes statistically significant. Two-way ANOVA with Bonferroni post-test. Data sets analyzed: wild type (WT), and the following DAT mutant residues: F587A, R588A, E589A, and K590A.

Table for Figure 4A

**2way ANOVA summary**	
1. Does mutation have the same effect at all values of mSIRK treatment?	Interaction accounts for 9.04% of the total variance.<br>F = 9.65. DFn=4 DFd=30<br>The P value is < 0.0001 (****)<br>If there is no interaction overall, there is a less than 0.01% chance of randomly observing so much interaction in an experiment of this size. The interaction is considered extremely significant.
2. Does mutation effect the result?	Mutation accounts for 6.34% of the total variance.<br>F = 27.06. DFn=1 DFd=30<br>The P value is < 0.0001 (****)<br>If mutation has no effect overall, there is a less than 0.01% chance of randomly observing an effect this big (or bigger) in an experiment of this size. The effect is considered extremely significant.
3. Does mSIRK treatment effect the result?	mSIRK treatment accounts for 77.59% of the total variance.<br>F = 82.79. DFn=4 DFd=30<br>The P value is < 0.0001 (****)<br>If mSIRK treatment has no effect overall, there is a less than 0.01% chance of randomly observing an this big (or bigger) in an experiment of this size. The effect is considered extremely significant

**Bonferroni post-test**<br>mSIRK treatment (WT vs F587A)	**Mean Diff.**	**95.00% CI of diff.**	**t**	**Summary**	**Adjusted P Value**
0	0	-62.52 to 59.51	0,06789	ns	P > 0.05
0,1	5,230	-55.79 to 66.25	0,2357	ns	P > 0.05
1	-52,61	-113.6 to 8.405	2,371	ns	P > 0.05
10	-166,4	-227.5 to -105.4	7,501	***	P<0.001
30	-42,76	-103.8 to 18.26	1,927	ns	P > 0.05

Table for Figure 4B

2way ANOVA summary	
1. Does mutation have the same effect at all values of mSIRK treatment?	Interaction accounts for 12.95% of the total variance.<br>F = 5.11. DFn=4 DFd=40<br>The P value = 0.0020 (**)<br>If there is no interaction overall, there is a 0.2% chance of randomly observing so much interaction in an experiment of this size. The interaction is considered very significant.
2. Does mutation effect the result?	Mutation accounts for 9.98% of the total variance.<br>F = 15.76. DFn=1 DFd=40<br>The P value = 0.0003 (***)<br>If mutation has no effect overall, there is a 0.029% chance of randomly observing an effect this big (or bigger) in an experiment of this size. The effect is considered extremely significant.
3. Does mSIRK treatment effect the result?	mSIRK treatment accounts for 51.75% of the total variance.<br>F = 20.43. DFn=4 DFd=40<br>The P value is < 0.0001 (****)<br>If mSIRK treatment has no effect overall, there is a less than 0.01% chance of randomly observing an this big (or bigger) in an experiment of this size. The effect is considered extremely significant.

**Bonferroni post-test**<br>mSIRK treatment (WT vs R588A)	**Mean Diff.**	**95.00% CI of diff.**	**t**	**Summary**	**Adjusted P Value**
0	0	-83.95 to 83.95	0	ns	P > 0.05
0,1	12,05	-71.90 to 96.00	0,3882	ns	P > 0.05
1	-29,29	-113.2 to 54.66	0,9436	ns	P > 0.05
10	-112,9	-196.8 to -28.93	3,636	**	P<0.01
30	-145,4	-229.4 to -61.46	4,684	***	P<0.001

Table for Figure 4C

**2way ANOVA summary**	
1. Does mutation have the same effect at all values of mSIRK treatment?	Interaction accounts for 1.38% of the total variance.<br>F = 2.16. DFn=4 DFd=30<br>The P value = 0.0978 (ns)<br>If there is no interaction overall, there is a 9.8% chance of randomly observing so much interaction in an experiment of this size. The interaction is considered not quite significant.
2. Does mutation effect the result?	Mutation accounts for <0.1% of the total variance.<br>F = 0.02. DFn=1 DFd=30<br>The P value = 0.8782 (ns)<br>If mutation has no effect overall, there is a 88% chance of randomly observing an effect this big (or bigger) in an experiment of this size. The effect is considered not significant.
3. Does mSIRK treatment effect the result?	mSIRK treatment accounts for 93.83% of the total variance.<br>F = 147.10. DFn=4 DFd=30<br>The P value is < 0.0001 (****)<br>If mSIRK treatment has no effect overall, there is a less than 0.01% chance of randomly observing an this big (or bigger) in an experiment of this size. The effect is considered extremely significant

**Bonferroni post-test**<br>mSIRK treatment (WT vs E589A)	**Mean Diff.**	**95.00% CI of diff.**	**t**	**Summary**	**Adjusted P Value**
0	0	-19.14 to 19.14	0	ns	P > 0.05
0,1	-7,443	-26.58 to 11.70	1,069	ns	P > 0.05
1	-13,57	-32.71 to 5.566	1,950	ns	P > 0.05
10	7,448	-11.69 to 26.59	1,070	ns	P > 0.05
30	11,16	-7.977 to 30.30	1,604	ns	P > 0.05

Table for Figure 4D

2way ANOVA summary	
1. Does mutation have the same effect at all values of AMPH treatment?	Interaction accounts for 0.90% of the total variance.<br>F = 1.02. DFn=4 DFd=40<br>The P value = 0.4106 (ns)<br>If there is no interaction overall, there is a 41% chance of randomly observing so much interaction in an experiment of this size. The interaction is considered not significant.
2. Does mutation effect the result?	Mutation accounts for 1.41% of the total variance.<br>F = 6.36. DFn=1 DFd=40<br>The P value = 0.0157 (p <0.05*)<br>If mutation has no effect overall, there is a 1.6% chance of randomly observing an effect this big (or bigger) in an experiment of this size. The effect is considered significant.
3. Does AMPH treatment effect the result?	AMPH treatment accounts for 88.79% of the total variance.<br>F = 99.89. DFn=4 DFd=40<br>The P value is < 0.0001 (****)<br>If AMPH treatment has no effect overall, there is a less than 0.01% chance of randomly observing an this big (or bigger) in an experiment of this size. The effect is considered extremely significant.

**Bonferroni post-test**<br>AMPH treatment (WT vs F587A)	**Mean Diff.**	**95.00% CI of diff.**	**t**	**Summary**	**Adjusted P Value**
0	0,0000	-75.27 to 75.27	0,0000	ns	P > 0.05
0,1	-8,078	-83.35 to 67.19	0,2903	ns	P > 0.05
1	-29,43	-104.7 to 45.84	1,057	ns	P > 0.05
10	-61,90	-137.2 to 13.37	2,224	ns	P > 0.05
30	-57,57	-132.8 to 17.70	2,068	ns	P > 0.05

Table for Figure 4E

2way ANOVA summary					
1. Does mutation have the same effect at all values of AMPH treatment?	Interaction accounts for 2.34% of the total variance.<br><br>F = 1.78. DFn=4 DFd=50<br><br>The P value = 0.1475 (ns)<br><br>If there is no interaction overall, there is a 15% chance of randomly observing so much interaction in an experiment of this size. The interaction is considered not significant.				
2. Does mutation effect the result?	Mutation accounts for 3.90% of the total variance.<br><br>F = 11.88. DFn=1 DFd=50<br><br>The P value = 0.0012 (**)<br><br>If mutation has no effect overall, there is a 0.12% chance of randomly observing an effect this big (or bigger) in an experiment of this size. The effect is considered very significant.				
3. Does AMPH treatment effect the result?	AMPH treatment accounts for 77.34% of the total variance.<br><br>F = 58.88. DFn=4 DFd=50<br><br>The P value is < 0.0001 (****)<br><br>If AMPH treatment has no effect overall, there is a less than 0.01% chance of randomly observing an this big (or bigger) in an experiment of this size. The effect is considered extremely significant.				
**Bonferroni post-test**<br>AMPH treatment (WT vs R588A)	**Mean Diff.**	**95.00% CI of diff.**	**t**	**Summary**	**Adjusted P Value**
0	0	-75.27 to 75.27	0	ns	P > 0.05
0,1	-12,90	-83.35 to 67.19	0,4065	ns	P > 0.05
1	-50,48	-104.7 to 45.84	1,590	ns	P > 0.05
10	-89,96	-137.2 to 13.37	2,834	*	P < 0.05
30	-91,31	-132.8 to 17.70	2,877	*	P < 0.05

Table for Figure 4F

2way ANOVA summary	
1. Does mutation have the same effect at all values of AMPH treatment?	Interaction accounts for 0.66% of the total variance.<br><br>F = 1.74. DFn=4 DFd=30<br><br>The P value = 0.1669 (ns)<br><br>If there is no interaction overall, there is a 17% chance of randomly observing so much interaction in an experiment of this size. The interaction is considered not significant.
2. Does mutation effect the result?	Mutation accounts for 0.66% of the total variance.<br><br>F = 7.04. DFn=1 DFd=30<br><br>The P value = 0.0126 (p<0.05*)<br><br>If mutation has no effect overall, there is a 1.3% chance of randomly observing an effect this big (or bigger) in an experiment of this size. The effect is considered significant.
3. Does AMPH treatment effect the result?	AMPH treatment accounts for 95.86% of the total variance.<br><br>F = 254.90. DFn=4 DFd=30<br><br>The P value is < 0.0001 (****)<br><br>If AMPH treatment has no effect overall, there is a less than 0.01% chance of randomly observing an this big (or bigger) in an experiment of this size. The effect is considered extremely significant

**Bonferroni post-test**<br>AMPH treatment (WT vs E589A)	**Mean Diff.**	**95.00% CI of diff.**	**t**	**Summary**	**Adjusted P Value**
0	0	-12.56 to 12.56	0	ns	P > 0.05
0,1	-1,871	-14.43 to 10.69	0,4097	ns	P > 0.05
1	7,225	-5.336 to 19.79	1,582	ns	P > 0.05
10	10,62	-1.943 to 23.18	2,324	ns	P > 0.05
30	11,12	-1.437 to 23.69	2,435	ns	P > 0.05

Table for Figure 4G

ANOVA summary	F (DFn, DFd)	P value
Treatment<br>Significant diff. among means (P < 0.05)? = yes	F =15.90	p<0.0008***

Tukey's multiple comparisons test	Mean Diff.	95.00% CI of diff.	Significant? P < 0.05?	Summary
basal vs mSIRK	-1,224	-1.872 to -0.5763	Yes	**
basal vs mSIRK + gallein	-0,07185	-0.7199 to 0.5762	No	ns
mSIRK vs mSIRK + gallein	1,153	0.4694 to 1.836	Yes	**

Unpaired t test					
	Mean Diff.	95.00% CI of diff.	T, df	P value	P value summary
DAT WT vs DAT R588A	1.203 ± 0.2806	0.5391 to 1.866	t=4.286, df=7	0.0036	**
